# Long-term open-label extension study of the safety and efficacy of intrathecal idursulfase-IT in patients with neuronopathic mucopolysaccharidosis II

**DOI:** 10.1016/j.ymgme.2022.07.016

**Published:** 2022-08-02

**Authors:** Joseph Muenzer, Barbara K. Burton, Paul Harmatz, Luis González Gutiérrez-Solana, Matilde Ruiz-Garcia, Simon A. Jones, Nathalie Guffon, Michal Inbar-Feigenberg, Drago Bratkovic, Michael Hale, Yuna Wu, Karen S. Yee, David A.H. Whiteman, David Alexanderian

**Affiliations:** a University of North Carolina at Chapel Hill, Chapel Hill, NC, USA; b Ann & Robert H. Lurie Children’s Hospital of Chicago, Northwestern University, Chicago, IL, USA; c UCSF Benioff Children’s Hospital Oakland, Oakland, CA, USA; d Infant Jesus Children’s Hospital, Madrid, Spain; e National Institute of Pediatrics, Mexico City, Mexico; f St Mary’s Hospital, Manchester University NHS Foundation Trust, University of Manchester, Manchester, UK; g Reference Center for Inherited Metabolic Diseases, Hospices Civils de Lyon, Lyon, France; h University of Toronto, Toronto, ON, Canada; i The Hospital for Sick Children, Toronto, ON, Canada; j Women’s and Children’s Hospital, North Adelaide, SA, Australia; k Takeda Development Center Americas, Inc., Cambridge, MA, USA; l Hale Scientific Statistics, LLC, Beaverton, OR, USA; m Takeda Development Center Americas, Inc., Lexington, MA, USA; n Affinia Therapeutics, Inc., Waltham, MA, USA

**Keywords:** Mucopolysaccharidosis II (MPS II), Cognitive impairment, Enzyme replacement therapy, Idursulfase, Intrathecal, Neuronopathic

## Abstract

Enzyme replacement therapy with weekly infused intravenous (IV) idursulfase is effective in treating somatic symptoms of mucopolysaccharidosis II (MPS II; Hunter syndrome). A formulation of idursulfase for intrathecal administration (idursulfase-IT) is under investigation for the treatment of neuronopathic MPS II. Here, we report 36-month data from the open-label extension (NCT02412787) of a phase 2/3, randomized, controlled study (HGT-HIT-094; NCT02055118) that assessed the safety and efficacy of monthly idursulfase-IT 10 mg in addition to weekly IV idursulfase on cognitive function in children older than 3 years with MPS II and mild-to-moderate cognitive impairment. Participants were also enrolled in this extension from a linked non-randomized sub-study of children younger than 3 years at the start of idursulfase-IT therapy. The extension safety population comprised 56 patients who received idursulfase-IT 10 mg once a month (or age-adjusted dose for sub-study patients) plus IV idursulfase (0.5 mg/kg) once a week. Idursulfase-IT was generally well tolerated over the cumulative treatment period of up to 36 months. Overall, 78.6% of patients had a least one adverse event (AE) related to idursulfase-IT; most treatment-emergent AEs were mild in severity. Of serious AEs (reported by 76.8% patients), none were considered related to idursulfase-IT treatment. There were no deaths or discontinuations owing to AEs. Secondary efficacy analyses (in patients younger than 6 years at phase 2/3 study baseline; *n* = 40) indicated a trend for improved Differential Ability Scale-II (DAS-II) General Conceptual Ability (GCA) scores in the early idursulfase-IT versus delayed idursulfase-IT group (treatment difference over 36 months from phase 2/3 study baseline: least-squares mean, 6.8 [90% confidence interval: −2.1, 15.8; *p* = 0.2064]). Post hoc analyses of DAS-II GCA scores by genotype revealed a clinically meaningful treatment effect in patients younger than 6 years with missense variants of the iduronate-2-sulfatase gene (*IDS*) (least-squares mean [standard error] treatment difference over 36 months, 12.3 [7.24]). These long-term data further suggest the benefits of idursulfase-IT in the treatment of neurocognitive dysfunction in some patients with MPS II. After many years of extensive review and regulatory discussions, the data were found to be insufficient to meet the evidentiary standard to support regulatory filings.

## 1. Introduction

Mucopolysaccharidosis II (MPS II; Hunter syndrome; OMIM 309900) is a rare lysosomal storage disease caused by deletion or pathogenic variants of the iduronate-2-sulfatase gene (*IDS*) [1,2]. The subsequent deficient enzymatic activity leads to accumulation of glycosaminoglycans (GAGs) in numerous tissues and organs. MPS II is chronic, progressive, and life-limiting [2,3]. All patients with MPS II are affected by somatic symptoms, which might include hepatosplenomegaly, abnormal facies, joint stiffness, skeletal deformity, cardiac disease, lung disease, communicating hydrocephalus, and hearing loss [4]. Approximately two-thirds of patients have the neuronopathic form of the disease and experience cognitive impairment in addition to somatic symptoms [5,6]. Evidence suggests that patients with cognitive impairment have a nearly fivefold higher risk of death than those without [7].

There is considerable heterogeneity among patients in the trajectory of neuronopathic MPS II: a typical profile might show a plateauing of cognitive development around the age of 3 years, followed by a deterioration from approximately 5 years of age [5,8,9]. Evidence suggests that the cognitive developmental course in patients with missense *IDS* genotypes differs from that in those with other null-type variants such as deletions or nonsense variants [8].

Enzyme replacement therapy (ERT) with intravenous (IV) idursulfase (Elaprase^®^, Takeda Pharmaceuticals USA, Inc., Lexington, MA, USA) is the standard of care for the treatment of the somatic symptoms of MPS II. In clinical trials and registry studies, IV infusions of idursulfase 0.5 mg/kg once a week were associated with significant benefits, including improvements in a composite endpoint incorporating measures of physical function and respiratory function and improved survival [3,7,10,11]. However, IV idursulfase does not cross the blood–brain barrier at therapeutic levels and has not been reported to affect cognitive decline in patients with neuronopathic disease [12]. Based on preclinical evidence showing that intrathecally administered idursulfase can penetrate into brain tissue [13], a formulation of idursulfase for intrathecal (IT) administration (idursulfase-IT) has been investigated for the treatment of neuronopathic MPS II.

A phase 2/3 randomized, controlled study (HGT-HIT-094; NCT02055118) assessed the efficacy of idursulfase-IT 10 mg once a month in addition to IV idursulfase once a week on cognitive function in children with MPS II and early cognitive impairment over 52 weeks [14]. Although the primary endpoint (change from baseline in the Differential Ability Scale-II [DAS-II] General Conceptual Ability [GCA] score at week 52) was not met, potential cognitive benefits of idursulfase-IT were indicated at week 52 in a prespecified analysis of patients who started treatment when they were younger than 6 years (least-squares mean [standard error] change from baseline in DAS-II GCA score at week 52 for idursulfase-IT vs no idursulfase-IT: −3.7 [2.9] vs −7.3 [4.2]). It is important to note that a decline in DAS-II GCA score over the course of the study does not necessarily imply a decline in cognitive function and instead may indicate a stabilization or an improvement in abilities. In addition, post hoc analyses revealed a clinically meaningful effect of idursulfase-IT on cognitive function at week 52 in patients younger than 6 years at baseline and with missense *IDS* variants (least-squares mean treatment difference, 16.1; 95% confidence interval [CI]: 3.3, 28.9; *p* = 0.0174). Idursulfase-IT was well tolerated over the 52 weeks.

Patients who completed the phase 2/3 trial as well as patients younger than 3 years from a linked open-label non-randomized sub-study of idursulfase-IT were enrolled in an ongoing open-label extension study (SHP609–302; NCT02412787), which is investigating the long-term safety and efficacy of monthly idursulfase-IT in addition to weekly IV idursulfase. Here, we present data on the safety and efficacy of idursulfase-IT in neuronopathic MPS II from this extension study for a total of up to 36 months of treatment.

## 2. Methods

### 2.1. Overview of analyses

**Fig. 1.**
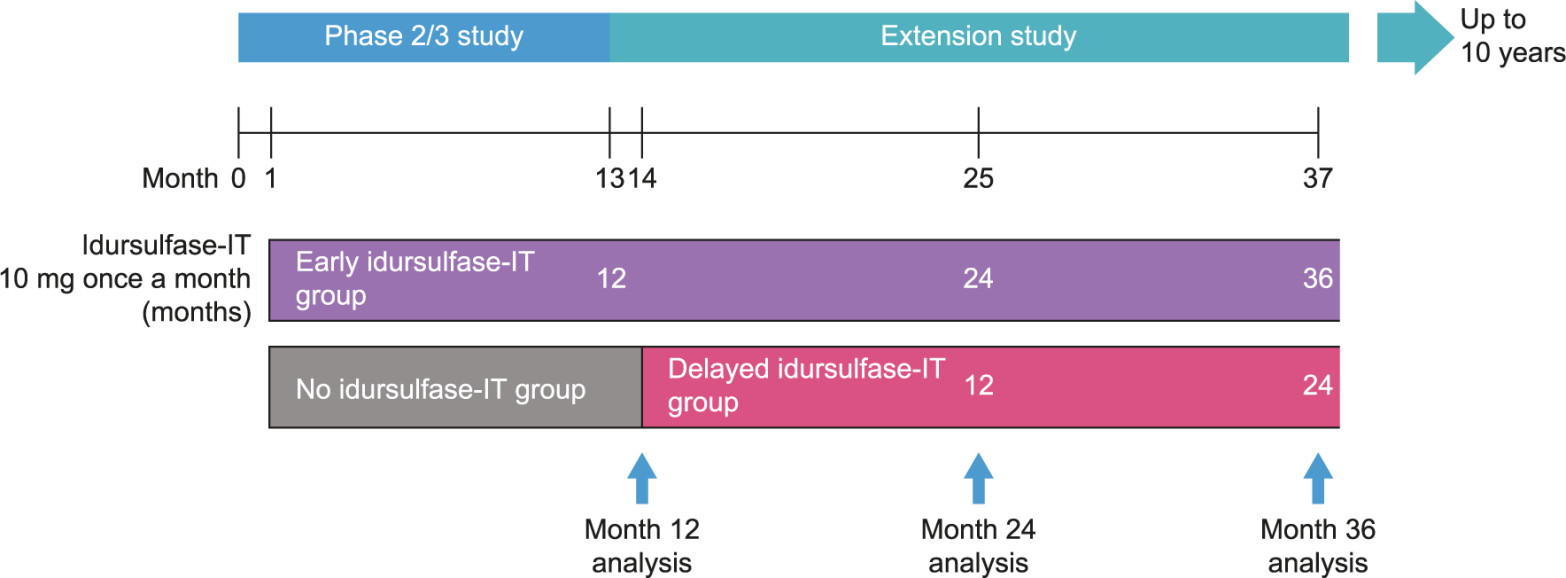
Overview of idursulfase-IT exposure and timing of analyses. Idursulfase-IT was administered via an IDDD or by lumbar puncture in the event of device malfunction. IDDD, intrathecal drug delivery device; IT, intrathecal.

We present an interim analysis after 24 months of treatment in the extension study, combined with the initial 12 months of idursulfase-IT or placebo during the randomized phase 2/3 study (Fig. 1).

The primary objective of this extension study was to evaluate the long-term safety of idursulfase-IT, with secondary analyses of efficacy by descriptive statistics. However, following discussion of data from the phase 2/3 study with the US Food and Drug Administration, it was decided that there was rationale for statistical analysis of efficacy data in the extension study beyond the planned summary description. Thus, an integrated summary of effectiveness was conducted to evaluate efficacy data over 36 months in the phase 2/3 study and extension combined, focusing on patients who were younger than 6 years at the phase 2/3 study baseline.

In the primary safety analyses reported here, baseline refers to the phase 2/3 study baseline for patients initially randomized to idursulfase-IT. For those randomized to placebo in the phase 2/3 study (and who subsequently received idursulfase-IT in the extension phase), baseline refers to the closest available assessment before the initial intrathecal drug delivery device (IDDD) implantation date, which took place at the start of the extension study. For secondary analyses of efficacy in the integrated summary of effectiveness, baseline refers simply to the phase 2/3 study baseline.

Following the identification in the phase 2/3 study of a subgroup of patients with a clinically relevant treatment response (those younger than 6 years at baseline with missense *IDS* variants), a post hoc analysis was conducted on the data from the extension study to assess the efficacy endpoints for patients younger than 6 years at baseline by *IDS* genotype (missense vs non-missense) [14].

### 2.2. Participants

Participants in the primary HGT-HIT-094 phase 2/3 study who completed assessments at week 52 and provided informed consent were eligible for inclusion in the extension phase. The inclusion and exclusion criteria for the initial phase 2/3 study are detailed elsewhere [14]. In brief, patients enrolled into the phase 2/3 study were boys aged between 3 and 18 years with a documented diagnosis of MPS II and evidence of MPS II-related cognitive impairment (based on age-dependent DAS-II GCA scores) [15]. All patients enrolled in the primary study were required to have received and tolerated a minimum of 4 months of treatment with IV idursulfase in the period immediately before screening [14]. Patients with an opening cerebrospinal fluid (CSF) pressure upon lumbar puncture of at least 30.0 cmH_2_O or a functioning CSF shunt device were excluded. Children who participated in a non-randomized sub-study of HGT-HIT-094 for patients younger than 3 years at baseline, all of whom received idursulfase-IT, were also eligible for inclusion in this extension study.

The first patient was enrolled into the extension study on April 14, 2015; patients were enrolled from 22 study sites in seven countries.

### 2.3. Study design

The primary phase 2/3 study was a 52-week, randomized controlled trial in which eligible patients were randomly assigned (2:1) to receive idursulfase-IT 10 mg once a month (i.e. every 28 days) or no idursulfase-IT, in addition to IV idursulfase 0.5 mg/kg once a week as standard of care. IV idursulfase was administered at least 48 h after idursulfase-IT. The design of the primary study is described in more detail separately [14]. The sub-study was a non-randomized, open-label, single-arm, 52-week study in which all patients received treatment with idursulfase-IT (dose adjusted based on reference brain weight by age).

In this ongoing, non-randomized extension study, all patients receive idursulfase-IT 10 mg once a month (i.e. every 28 days), except those aged >8 to 30 months at dosing, who receive idursulfase-IT 7.5 mg instead. All patients continue to receive weekly IV idursulfase (Fig. 1). The extension study protocol permits same-day administration of IV idursulfase and idursulfase-IT.

During the primary and extension studies, idursulfase-IT was administered via the SOPH-A-PORT^®^ Mini S, Implantable Access Port, Spinal, Mini Unattached, with Guidewire (SOPH-A-PORT Mini S; Sophysa SA, Orsay, France) IDDD [14]. If the intrathecal space was inaccessible via the IDDD or there were other mechanical complications with the device, the study drug was delivered via lumbar puncture.

The study was approved by the relevant institutional review boards/institutional ethics committees and was conducted in compliance with the International Conference on Harmonisation Good Clinical Practice guidelines and the Declaration of Helsinki. For all patients, written informed consent was obtained from the parent(s) or legally authorized guardian(s); assent from the patient was also acquired, if applicable.

### 2.4. Treatment groups

In the extension study, treatment groups were defined according to the randomized treatment assignment in the phase 2/3 study: the early idursulfase-IT group comprised patients who received idursulfase-IT in the phase 2/3 trial, whereas the delayed idursulfase-IT group comprised those who did not receive idursulfase-IT before the extension. The extension study is ongoing.

Two interim analyses were planned (Fig. 1). The first was scheduled for month 25, at which point the early idursulfase-IT group and the delayed idursulfase-IT group had received idursulfase-IT treatment for 24 months and 12 months, respectively, or had discontinued. The second was scheduled for month 37, at which point the early idursulfase-IT group and the delayed idursulfase-IT group had received idursulfase-IT treatment for 36 months and 24 months, respectively, or had discontinued (Fig. 1). Each group had a period of 1 month allocated to surgical IDDD implantation and recovery before the initiation of idursulfase-IT. Based on the duration of observations from the start of the primary study, the extension baseline and two interim analyses are hereafter referred to as the month 12, month 24, and month 36 analyses, respectively.

### 2.5. Assessments and endpoints

Safety endpoints included adverse events (AEs), changes in clinical laboratory values, vital signs, 12‑lead electrocardiogram (ECG) recordings, brain magnetic resonance imaging parameters, CSF assessments (including chemistries, cell counts, and iduronate-2-sulfatase [I2S] concentration), and anti-idursulfase antibodies (including neutralizing antibodies, NAbs) in the CSF and serum.

An important secondary efficacy endpoint was change from baseline in DAS-II GCA scores (early years battery). Other secondary efficacy endpoints included changes from baseline in DAS-II standard cluster and composite scores (early years battery), and Vineland Adaptive Behavior Scales-II (VABS-II) Adaptive Behavior Composite (ABC) and domain standard scores. Exploratory efficacy endpoints were ordered categorical outcomes (three categories: above-average, average, or below-average cognitive development) for DAS-II GCA scores (early years battery).

Pharmacodynamic endpoints included changes from baseline in total GAGs and heparan sulfate (HS) levels in the CSF, samples of which were collected via the IDDD or lumbar puncture. Details of the assay methodology are reported elsewhere [14].

### 2.6. Statistical methods

All descriptive summary analyses of safety and efficacy data were based on the safety population, defined as all patients in the extension study who underwent IDDD implantation surgery and/or received at least one dose (full or partial) of study drug. This included patients younger than 3 years at baseline who were enrolled in the sub-study; these patients were included in the early idursulfase-IT group. Patients in the sub-study did not have baseline DAS-II GCA scores and, therefore, were not included in the efficacy analysis (the data from the sub-study will be reported separately). Efficacy data are reported for patients younger than 6 years at phase 2/3 study baseline and for the subgroup of patients younger than 6 years at phase 2/3 study baseline with missense *IDS* variants (post hoc analysis).

To assess treatment exposure, a percentage was calculated by IDDD and lumbar puncture separately for each patient, from which the mean and standard deviation (SD) were determined.

All statistical analyses were performed using the Statistical Analysis System software version 9.3 or higher (SAS Institute, Cary, NC, USA).

#### 2.6.1. Prespecified analyses

The efficacy analysis compared change from baseline in DAS-II GCA scores in the early idursulfase-IT group with that in the delayed idursulfase-IT group using a mixed-effects model for repeated measures (MMRM) analysis. The model included fixed categorical effects for treatment, visit week, treatment by visit week interaction, and baseline DAS-II GCA classification factor (either ≤70 or > 70). Weeks 16 and 40 of the extension for the delayed idursulfase-IT group were not included in the MMRM analysis owing to the lack of matching visits in the early idursulfase-IT group. Changes from baseline in DAS-II standard cluster and composite scores and VABS-II ABC scores were also evaluated with MMRM analysis. An exploratory, ordered, categorical analysis was conducted for DAS-II GCA scores at the month 12, month 24, and month 36 analyses (prespecified at month 24 only) for cognitive development categories of above average, average, or below average. A rate-of-change (weighted slope) analysis was also performed on DAS-II GCA and VABS-II ABC scores to explore the treatment effect over 36 months (further details on the weighted analysis methodology can be found in the primary study paper [14]).

Other efficacy endpoints are summarized descriptively, with statistical model estimates of least-squares means, treatment differences, *p* values, and 90% CIs provided where appropriate; statistical tests were two-sided and performed at the 0.1 level of significance.

#### 2.6.2. Post hoc analyses

Post hoc analyses for patients younger than 6 years at phase 2/3 study baseline with missense *IDS* variants were conducted (using an MMRM) to assess the changes from baseline in DAS-II GCA and VABS-II ABC scores in the early idursulfase-IT and delayed idursulfase-IT groups in the subgroups of patients younger than 6 years with missense and non-missense *IDS* genotypes.

## 3. Results

### 3.1. Patient population

The safety population comprised 56 patients. This included 47 out of 49 patients (95.9%) who completed the 52-week phase 2/3 study and were enrolled in the extension study (early idursulfase-IT group, *n* = 32; delayed idursulfase-IT group, *n* = 15; two patients [4.1%] did not complete the phase 2/3 study owing to withdrawal of consent); plus, nine patients who had participated in the open-label sub-study (data for these patients were included in the early idursulfase-IT group for safety and exposure analyses). At the month 36 analysis, 54 patients were participating in the extension study; two patients (early idursulfase-IT group) discontinued owing to withdrawal of consent. The efficacy analysis population (patients younger than 6 years at phase 2/3 study baseline) included 40 patients (early idursulfase-IT, *n* = 28; delayed idursulfase-IT, *n* = 12). Of these, 19 had missense *IDS* variants (early idursulfase-IT, *n* = 13; delayed idursulfase-IT, *n* = 6).

Demographics and baseline characteristics for the safety and efficacy populations are presented in Table 1. Mean (SD) age at baseline for the safety population was 4.9 (2.1) years; for the efficacy population of patients younger than 6 years at baseline, mean (SD) age was 4.3 (0.7) years. The most common *IDS* variant type was missense (in 46.4% and 47.5% in the safety and efficacy populations, respectively). Detail on demographics and baseline characteristics for the patients with missense *IDS* variants versus those with other *IDS* genotypes have been described in the primary study publication [14].

### 3.2. Treatment exposure

In the safety population, the median (range) number of doses received was 47.5 (27–68) and the median (range) duration of treatment was 43.6 (24.4–62.4) months. The mean (SD) number of idursulfase-IT injections received by the safety population was 47.8 (10.3). The mean (SD) percentage of doses received via IDDD was 80.1% (27.6%) and by lumbar puncture was 28.4% (32.8%).

### 3.3. Safety and immunogenicity

Long-term treatment with idursulfase-IT for up to 36 months was generally well tolerated, consistent with the findings of the phase 2/3 study. Approximately three-quarters of patients (78.6%) reported AEs related to idursulfase-IT (Table 2); overall, most treatment-emergent AEs (TEAEs) were mild in severity. In the early idursulfase-IT group, the most common TEAEs considered by the investigator to be related to idursulfase-IT were vomiting (58.5% of patients), headache (34.1%), and pyrexia (29.3%); in the delayed idursulfase-IT group, these were increased CSF white blood cell count (26.7%), decreased body temperature, headache, vomiting, increased systolic blood pressure, and increased diastolic blood pressure (20.0% each). Serious AEs (SAEs) were reported by 80.5% of patients in the early idursulfase-IT group and 66.7% of patients in the delayed idursulfase-IT group; none of the SAEs were considered by the investigator to be related to idursulfase-IT. No deaths or discontinuations due to AEs were reported. There were no apparent safety concerns with same-day administration of idursulfase-IT and IV idursulfase.

Most patients who received idursulfase-IT reported at least one IDDD-related event (91.1%; Table 2): 23 patients (41.1%) had a total of 29 IDDD failures and 18 patients (32.1%) had a total of 18 IDDD malfunctions. The most common reason for failure or malfunction was inability to aspirate CSF before dose administration (reported by 30 patients [53.6%]; 68 events involving 40 IDDDs). Of those who received at least one dose of idursulfase-IT via IDDD (*n* = 31), 26 patients reported a total of 236 TEAEs that were deemed by the investigator as being associated with the treatment administration process. Of the 25 patients who received at least one idursulfase-IT dose via lumbar puncture, 13 reported 114 TEAEs that were deemed by the investigator as being associated with the administration process.

Aside from those noted above, no clinically significant changes from baseline in CSF laboratory values (chemistries, cell counts, and I2S concentration) or clinical laboratory values (hematology, serum chemistry, and urinalysis) or ECG values were observed during the study up to the month 36 analysis.

Most patients had positive serum anti-idursulfase antibody results at phase 2/3 study baseline (67.9%). Of the 18 patients who did not, nine developed first detectable serum anti-idursulfase antibodies during the extension (these were transient in five patients). Nearly half of patients (46.4%) had positive serum NAb results at phase 2/3 study baseline, and 69.6% had positive serum NAb results at post-baseline time points. All patients either already had positive CSF anti-idursulfase antibody results at baseline (44.6%) or remained negative for CSF anti-idursulfase antibodies throughout the study. Three patients (5.4%) had positive CSF NAb status at baseline, compared with 14 (25%) at post-baseline time points.

### 3.4. Efficacy

#### 3.4.1. Change from baseline in DAS-II GCA scores in patients younger than 6 years

**Fig. 2.**
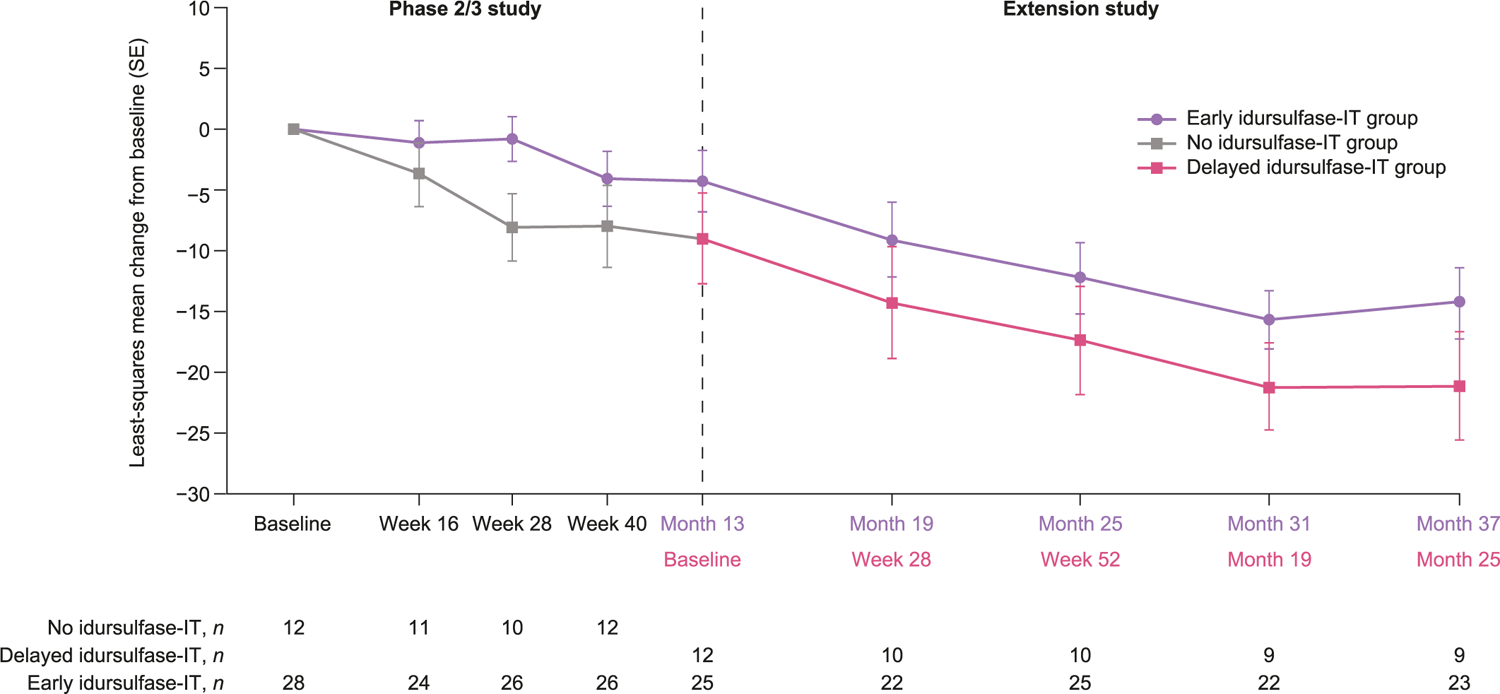
Least-squares mean change from baseline in DAS-II early years GCA scores in patients younger than 6 years at baseline over 36 months. Baseline is phase 2/3 study baseline. DAS-II, Differential Ability Scales-II; GCA, General Conceptual Ability; IT, intrathecal; SE, standard error.

**Fig. 3.**
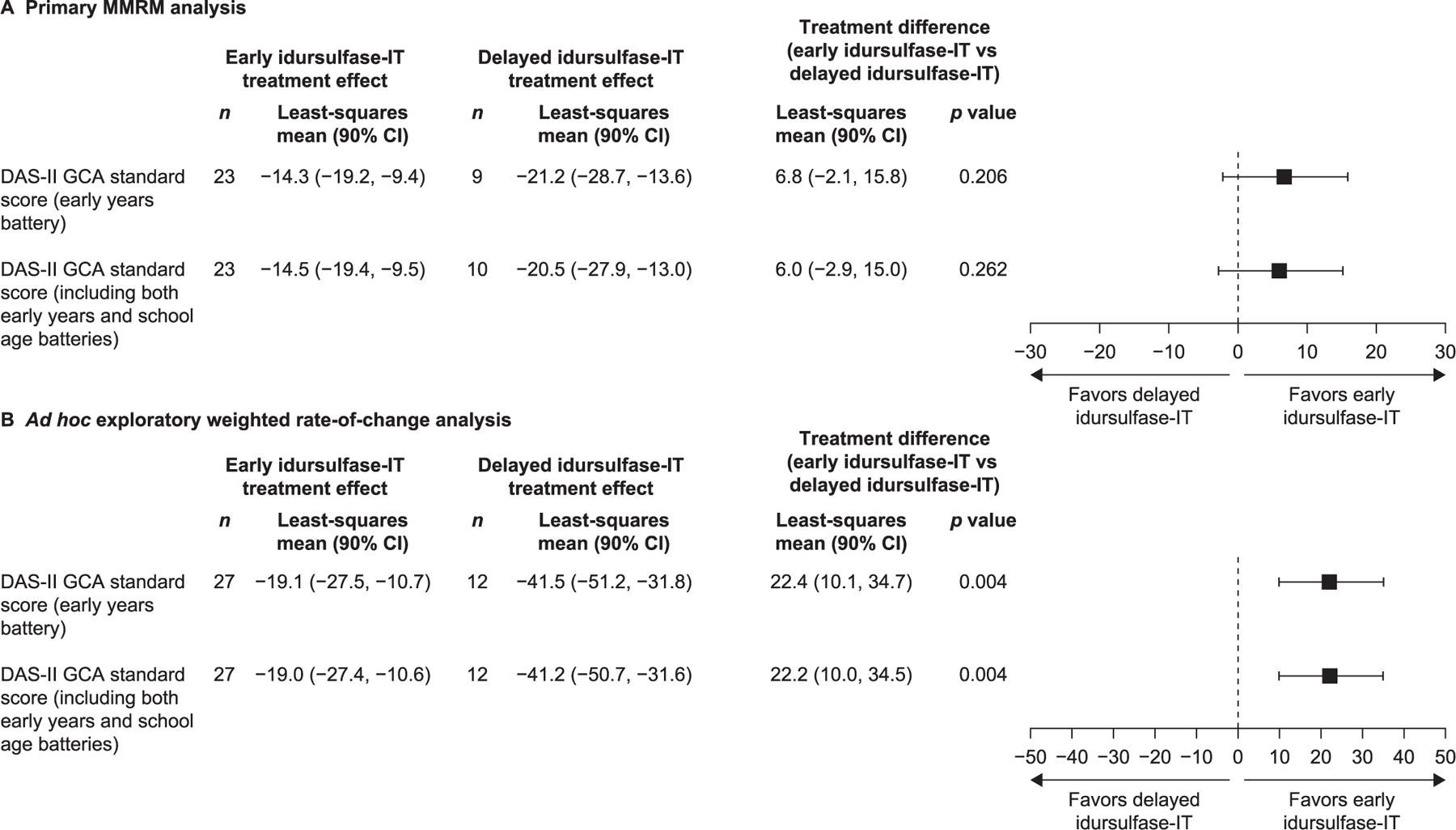
Change from baseline in DAS-II GCA scores at month 36 in patients younger than 6 years at baseline. For the weighted rate-of-change analysis at month 36, the weighted slope was corrected for floor effect. The early years battery includes children aged 2 years 6 months to 6 years 11 months; the school age battery includes children aged 7 years to 17 years 11 months. CI, confidence interval; DAS-II, Differential Ability Scales-II; GCA, General Conceptual Ability; IT, intrathecal; MMRM, mixed-effects model for repeated measures; SE, standard error.

DAS-II GCA scores from the early years battery continued to decline from baseline throughout the extension study, in both the early and delayed idursulfase-IT treatment groups (Fig. 2). At month 36, the least-squares mean (90% CI) change from baseline in DAS-II GCA score by MMRM analysis was −14.3 (−19.2, −9.4) for the early idursulfase-IT group and −21.2 (−28.7, −13.6) for the delayed idursulfase-IT group (Fig. 3A). The difference (90% CI) between the early and delayed idursulfase-IT groups (6.8 [−2.1, 15.8], *p* = 0.2064) was similar to that reported at the end of the phase 2/3 trial [14], in which the reduction from baseline was numerically smaller in the idursulfase-IT group than in the no idursulfase-IT group (for the current analyses defined as the early idursulfase-IT and delayed idursulfase-IT groups, respectively). Exploratory weighted rate-of-change analysis of change from baseline in DAS-II GCA scores at month 36 also favored early idursulfase-IT treatment (Fig. 3B). A trend towards a potential benefit in the DAS-II cluster scores was also observed for the early idursulfase-IT treatment group at month 36 (Supplementary Fig. 1).

#### 3.4.2. Exploratory prespecified analysis (ordered categorical outcomes in DAS-II GCA scores) in patients younger than 6 years

**Fig. 4.**
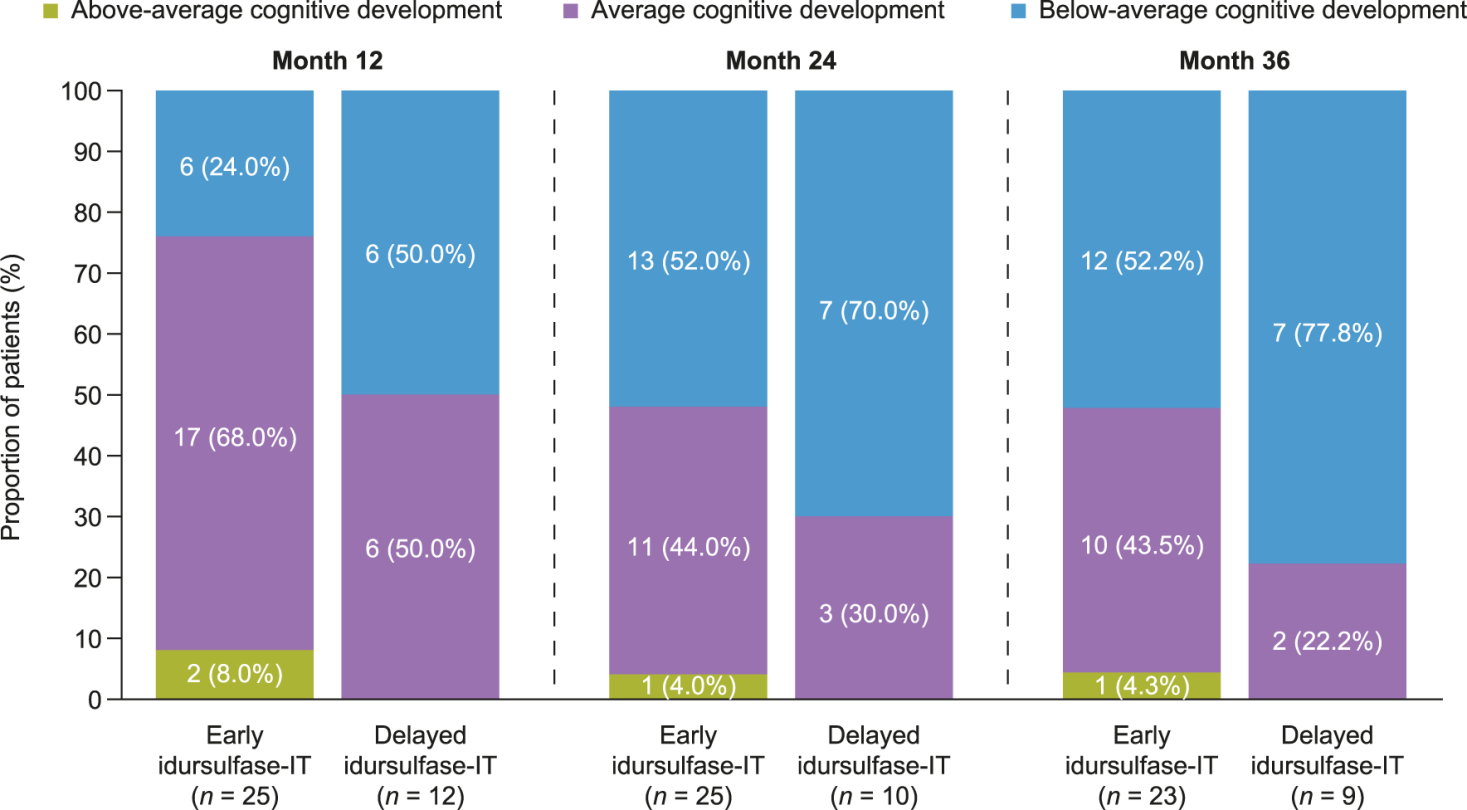
Ordered categorical outcomes in DAS-II GCA scores from the early years battery at months 12, 24, and 36 in patients younger than 6 years at phase 2/3 study baseline. Results for 12-month analyses are based on ordinal logistic regression. Categories were defined for 24-month time point as follows: above average = GCA at month 24 > 10 points higher than GCA at month 12; average = GCA at month 24 within 10 points of GCA at month 12; below average = GCA at month 24 > 10 points lower than GCA at month 12. Analyses at months 12 and 36 were not defined in the protocol or statistical analysis plan. Note that the 10-point difference becomes increasingly stringent as the study duration increases. DAS-II, Differential Ability Scales-II; GCA, General Conceptual Ability; IT, intrathecal.

There was a non-significant trend for improved outcomes in patients in the early idursulfase-IT compared with the delayed idursulfase-IT group in an analysis of ordered categorical outcomes in DAS-II GCA scores from the early years battery. At month 24, the proportions of patients with average or above-average cognitive development were 48.0% (12/25) and 30.0% (3/10), respectively. Although these proportions were lower than those at month 12 (76.0% and 50.0%, respectively), there was little change from months 24 to 36, when these proportions were 47.8% and 22.2%, respectively (Fig. 4).

#### 3.4.3. Change from baseline in DAS-II GCA scores in patients younger than 6 years by genotype (post hoc analyses)

**Fig. 5.**
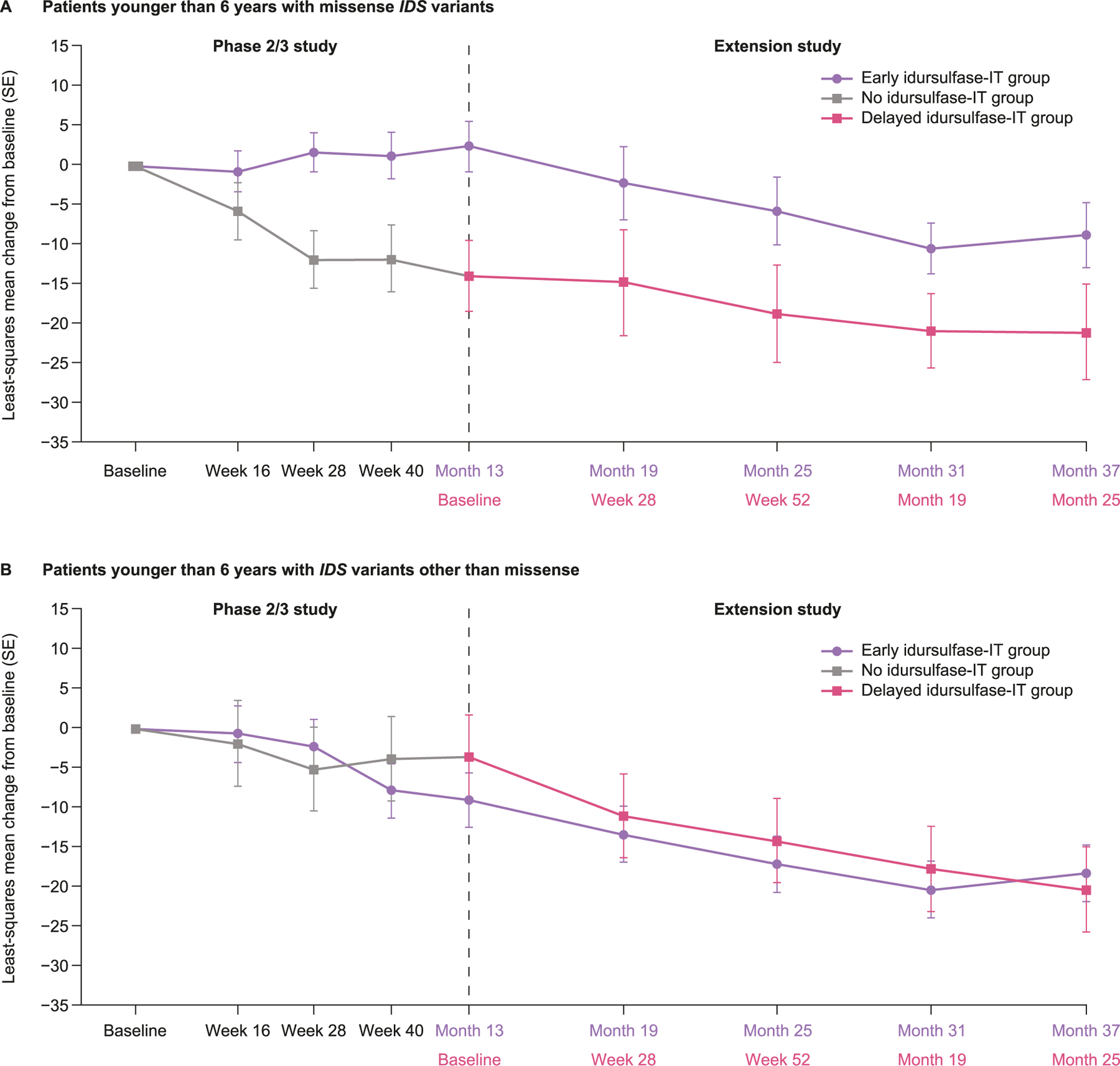
Least-squares mean change from baseline in DAS-II GCA scores in patients younger than 6 years at baseline. Baseline is phase 2/3 study baseline. DAS-II, Differential Ability Scales-II; GCA, General Conceptual Ability; *IDS*, iduronate-2-sulfatase gene; IT, intrathecal; SE, standard error.

For patients younger than 6 years at baseline with missense *IDS* variants, there was a notably smaller decline from baseline in DAS-II GCA scores for the early idursulfase-IT group compared with the delayed idursulfase-IT group over 36 months (Fig. 5A). At the month 24 analysis, the least-squares mean (standard error [SE]) treatment difference was 12.9 (7.6). This difference was maintained at the month 36 analysis (least-squares mean [SE] treatment difference, 12.3 [7.2]). Treatment differences for DAS-II cluster scores in patients younger than 6 years at baseline with missense *IDS* genotype also showed trends in favor of early idursulfase-IT treatment (Supplementary Fig. 2A).

In patients with *IDS* variants other than missense, there was no clear difference between the early idursulfase-IT and the delayed idursulfase-IT treatment groups in the changes from baseline in DAS-II GCA scores over 36 months (Fig. 5B), nor in DAS-II cluster scores (Supplementary Fig. 2B).

#### 3.4.4. Change from baseline in VABS-II ABC scores in patients younger than 6 years

There were no clear trends in treatment group differences for VABS-II ABC scores after 24 and 36 months in patients younger than 6 years at baseline, regardless of genotype (Supplementary Table 1).

#### 3.4.5. CSF total GAG and HS levels in patients younger than 6 years

In the overall subgroup of patients younger than 6 years, CSF total GAG levels were reduced from baseline (mean [SD]) by −71.9% (13.8) and −70.3% (15.7) at months 24 and 36, respectively, for early idursulfase-IT; similar reductions were reported at the same time points for delayed idursulfase-IT (mean [SD]: −73.9% [10.3] and −76.5 [10.2]). Reductions from baseline in HS levels were reported at months 24 and 36 (mean [SD]) with early idursulfase-IT (−22.4% [54.9] and −14.8 [36.3], respectively) and with delayed idursulfase-IT (−38.6% [22.8] and −32.6 [22.4], respectively); however, the data were more variable than those for CSF total GAG levels with evidence of a gradual reversal towards baseline levels.

**Fig. 6.**
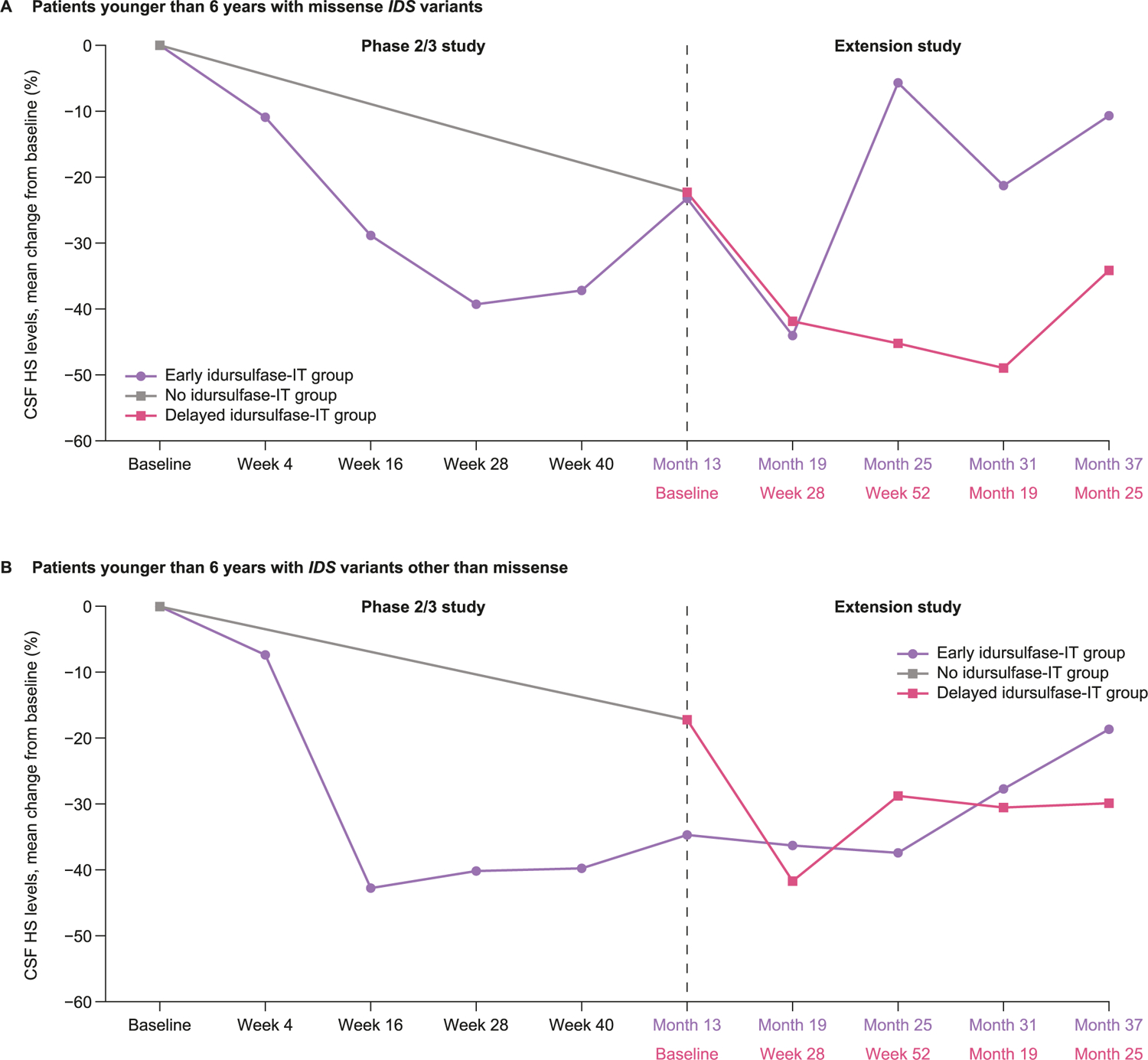
Mean percentage change from baseline in CSF HS levels in patients younger than 6 years at baseline with (A) missense *IDS* variants and (B) any *IDS* variant other than missense. CSF, cerebrospinal fluid; HS, heparan sulfate; *IDS*, iduronate-2-sulfatase gene; IT, intrathecal.

There was also a decrease in CSF total GAG levels in response to initiation of idursulfase-IT in the subgroup of patients with missense *IDS* variants; in patients with other *IDS* genotypes, CSF total GAG levels initially increased slightly before showing reductions similar to those in the missense subgroup (Supplementary Fig. 3). There was an initial decrease in CSF HS levels following initiation of idursulfase-IT, although as noted above, these data were more variable than those for GAG levels (Fig. 6).

## 4. Discussion

This interim analysis of data from the open-label extension study has demonstrated that idursulfase-IT 10 mg once a month, in addition to IV idursulfase 0.5 mg/kg once a week, was generally well tolerated during treatment periods of up to 36 months. The performance of the IDDD was acceptable, with some patients requiring additional surgical management; patients could also receive idursulfase-IT via lumbar puncture. The rates of AEs and other safety findings were consistent with those reported in the primary phase 2/3 study.

Efficacy analyses were conducted in patients younger than 6 years at phase 2/3 study baseline; in that study, there was a more pronounced treatment response with idursulfase-IT in this subgroup than in the overall population. In this extension study, cognitive function (assessed by DAS-II GCA scores) continued to decrease from baseline in both the early idursulfase-IT and the delayed idursulfase-IT treatment groups over 36 months. However, the (non-significant) difference in DAS-II GCA scores in favor of idursulfase-IT observed at the end of the phase 2/3 trial after 1 year of treatment was maintained throughout the 36-month extension period for the early idursulfase-IT versus the delayed idursulfase-IT treatment groups. Furthermore, the post hoc subgroup of patients younger than 6 years at baseline and with missense *IDS* variants, in which there was a significant and clinically meaningful treatment effect of idursulfase-IT versus no idursulfase-IT on cognitive function in the phase 2/3 study, also demonstrated a sustained difference in DAS-II GCA scores between the early idursulfase-IT and delayed idursulfase-IT treatment groups during the extension. In pharmacodynamic analyses, the reductions in CSF total GAG levels observed in response to initiation of idursulfase-IT in the phase 2/3 study were sustained throughout the extension phase, whereas the trend for partial reversal of initial reductions of CSF HS levels continued with ongoing treatment. This unexplained drop in CSF HS levels may have been dose-related and might help to explain why the cognitive results are not more pronounced.

These data, along with those from the primary study [14], support the benefits of early initiation of ERT in patients with MPS II; further evidence for this approach includes preclinical and clinical evidence with IV idursulfase and real-world outcomes data, plus case reports of siblings facilitating comparisons of early versus delayed treatment [16–18]. Based on the understanding of the natural history of MPS II, it might be expected that most patients aged from 3 to 6 years may have reached a plateau of cognitive development without yet experiencing appreciable loss of developmental milestones and skills already acquired [8]. This is an important window of therapeutic opportunity for patients with neuronopathic MPS II; notably, it has been proposed that treatment should ideally be initiated before a child reaches the plateau in cognitive development [8]. However, it is important to note that more than half of the patients included in the efficacy analysis (and, therefore, under 6 years of age) had baseline DAS-II GCA scores of no more than 70. Variations between patients with neuronopathic MPS II for the ages at which these milestones are reached create a challenging setting in which to demonstrate therapeutic benefits in clinical trials. It is important to acknowledge the real-world challenges in ensuring that treatment for MPS II is started early enough. In the absence of a family history to raise suspicion of MPS II, newborn screening is likely to be the only means of achieving a diagnosis and treatment initiation sufficiently early to optimize therapeutic benefit. Furthermore, the need for early treatment emphasizes the importance of being able to predict MPS II phenotype to identify those patients likely to benefit from idursulfase-IT treatment.

Stabilization of the DAS-II GCA score is equivalent to normal development and does not just indicate retention of cognitive ability. Some decline in DAS-II GCA scores can still be consistent with improvement or acquisition of new cognitive skills. A decline in GCA scores does not necessarily mean that the individual has lost skills. In the phase 2/3 trial, investigators’ and families’ impressions were that, during the study time frame, the patients did not lose skills but typically gained skills at a less than normal rate [14]. Thus, the fact that changes from baseline in DAS-II GCA scores in the early idursulfase-IT group were numerically smaller than in the delayed idursulfase-IT group may suggest a benefit of earlier treatment initiation. Similarly, the observed reduction in proportions of patients with average or above-average cognitive development between months 12 and 24 in the categorical analysis may be explained by development continuing at a slightly slower pace in children with MPS II than in typically developing children. If one presumes that the damage to brain development precedes symptom expression, it might be difficult to keep up the rapid pace of early development.

A post hoc analysis showed that the clinically meaningful treatment effect observed in patients younger than 6 years with missense *IDS* variants in the phase 2/3 study was maintained over at least 36 months in the extension study. For those with other genotypes, changes from baseline were generally unaffected by the initial randomization group during the phase 2/3 study and extension; however, as discussed in the primary paper [14], there were individual exceptions to this. Missense is the most common *IDS* variant type in MPS II, and a significant proportion of patients with missense *IDS* variants (up to 79%) have neuronopathic disease [19,20]. There is a plausible rationale for enhanced response to idursulfase-IT in patients with missense *IDS* genotypes. As discussed in the primary study publication [14], a possible explanation could be that the presence of residual endogenous I2S in patients with missense *IDS* variants may reduce the likelihood of developing anti-idursulfase antibodies compared with patients with other pathogenic variants that cause deficient activity of I2S [8,21]. The presence of anti-idursulfase NAbs has been associated with a reduced treatment benefit [22]. Also, administration of idursulfase-IT may supplement the residual enzyme activity in these patients, providing a sufficient level of enzyme to reduce lysosomal accumulation of GAGs [14].

This study had a number of limitations. All patients in this open-label extension phase were receiving idursulfase-IT, and treatment groups were differentiated only by the duration of treatment by virtue of randomized assignment in the original study. In the efficacy analysis population, the mean between-groups change from baseline in DAS-II GCA scores did not reach statistical significance in the MMRM analysis at month 36. The more notable differences between treatment groups in the subset of patients with missense *IDS* variants need to be interpreted with caution, because they were post hoc analyses not specified in the original protocol. The original phase 2/3 study was subject to strict inclusion criteria, which may limit the generalizability of the data to other patients with neuronopathic MPS II. Furthermore, there is limited potential to place the current findings in context owing to the scarcity of natural history data available on neuronopathic MPS II. The dose selected for this study was based on dose–response data from a phase 1 study; however, given the results of this study, it would be of interest to explore the effects on clinical outcomes of higher doses and/or a more frequent dosing interval.

These limitations notwithstanding, these interim findings were consistent with the results from the phase 2/3 study, thus strengthening and extending the previous conclusions.

## 5. Conclusions

In patients with neuronopathic MPS II, long-term idursulfase-IT was generally well tolerated; the safety and tolerability profile over 36 months of treatment is consistent with that reported in the 52-week phase 2/3 trial [14]. The smaller decline in DAS-II GCA scores from baseline with idursulfase-IT versus no idursulfase-IT observed after 52 weeks [14] was maintained throughout the extension study once all patients were receiving idursulfase-IT. Post hoc analyses of DAS-II GCA scores by genotype revealed that the significant and clinically meaningful treatment effect on DAS-II GCA scores in patients younger than 6 years with missense *IDS* variants, observed in the phase 2/3 study, was also maintained over 36 months. After many years of extensive review and regulatory discussions, the data were found to be insufficient to meet the evidentiary standard to support regulatory filings. Idursulfase-IT will continue to be made available to patients who are currently enrolled in the ongoing open-label extension studies until another approved treatment is available to address the cognitive symptoms.

## Supplementary Material

Supplementary data

## Figures and Tables

**Table 1:** Demographics and baseline^a^ characteristics for the safety and efficacy analysis populations.

	Safety analysis population^b^			
	
	Early idursulfase-IT (*n* = 32)	Delayed idursulfase-IT (*n* = 15)	Sub-study^c^ (*n* = 9)	Overall (*N* = 56)

Age at baseline, years				
Mean (SD)	4.9 (1.4)	6.3 (2.7)	2.5 (0.5)	4.9 (2.1)
Median (range)	4.6 (3.1, 8.7)	5.7 (4.1, 14.3)	2.6 (1.4, 3.0)	4.5 (1.4, 14.3)
Race, *n* (%)				
Asian	4 (12.5)	0	0	4 (7.1)
Black or African American	1 (3.1)	0	0	1 (1.8)
White	21 (65.6)	12 (80.0)	8 (88.9)	41 (73.2)
Other	6 (18.8)	3 (20.0)	1 (11.1)	10 (17.9)
Number of patients per type of *IDS* variant, *n* (%)				
Missense	15 (46.9)	7 (46.9)	4 (44.4)	26 (46.4)
Nonsense	3 (9.4)	1 (6.7)	1 (11.1)	5 (8.9)
Frameshift	5 (15.6)	3 (20.0)	0	8 (14.3)
Large deletion or complete deletion/large rearrangement	5 (15.6)	0	3 (33.3)	8 (14.3)
Intronic mutation	2 (6.3)	2 (13.3)	1 (11.1)	5 (8.9)
Splice site	1 (3.1)	0	0	1 (1.8)
Unclassifiable	1 (3.1)	2 (13.3)	0	3 (5.4)
Height, cm				
Mean (SD)	111.7 (9.5)	118.1 (11.2)	93.7 (5.5)	110.8 (12.1)
Median (range)	109.4 (95.7, 140.0)	115.0 (100.0, 144.4)	93.5 (85.4, 101.6)	110.0 (85.4, 144.4)
Weight, kg				
Mean (SD)	24.5 (5.0)	28.0 (8.2)	17.8 (3.3)	24.4 (6.6)
Median (range)	23.8 (18.5, 39.8)	26.9 (19.4, 48.7)	16.9 (14.2, 23.9)	23.3 (14.2, 48.7)
Number of patients per baseline DAS-II GCA score category, *n* (%)				
DAS-II GCA score ≤ 70	20 (62.5)	13 (86.7)	NA	33 (58.9)
DAS-II GCA score > 70	12 (37.5)	2 (13.3)	NA	14 (25.0)
	Efficacy analysis population^d^			
	
	Early idursulfase-IT (*n* = 28)	Delayed idursulfase-IT (*n* = 12)		Overall (*N* = 40)

Age at baseline, years				
Mean (SD)	4.3 (0.7)	4.2 (0.9)		4.3 (0.7)
Median (range)	4.4 (3.1, 5.7)	3.9 (3.1, 5.9)		4.3 (3.1, 5.9)
Race, *n* (%)				
Asian	3 (10.7)	0		3 (7.5)
Black or African American	1 (3.6)	0		1 (2.5)
White	19 (67.9)	9 (75.0)		28 (70.0)
Other	5 (17.9)	3 (25.0)		8 (20.0)
Number of patients per type of *IDS* variant, *n* (%)				
Missense	13 (46.4)	6 (50.0)		19 (47.5)
Nonsense	2 (7.1)	0		2 (5.0)
Frameshift	5 (17.9)	2 (16.7)		7 (17.5)
Large deletion or complete deletion/large rearrangement	5 (17.9)	0		5 (12.5)
Intronic	2 (7.1)	2 (16.7)		4 (10.0)
Splice site	1 (3.6)	0		1 (2.5)
Unclassifiable	0	2 (16.7)		2 (5.0)
Height, cm				
Mean (SD)	108.6 (5.9)	106.0 (6.4)		107.8 (6.06)
Median (range)	107.0 (95.7, 122.1)	105.2 (93.0, 118.8)		106.8 (93.0, 122.1)
Weight, kg				
Mean (SD)	23.2 (3.2)	22.0 (4.0)		22.9 (3.5)
Median (range)	23.1 (18.5, 31.9)	21.9 (17.0, 30.8)		23.0 (17.0, 31.9)
Number of patients per baseline DAS-II GCA score category, *n* (%)				
DAS-II GCA score ≤ 70	16 (57.1)	7 (58.3)		23 (57.5)
DAS-II GCA score > 70	12 (42.9)	5 (41.7)		17 (42.5)

DAS-II, Differential Ability Scales-II; GCA, General Conceptual Ability; IDDD, intrathecal drug delivery device; *IDS*, iduronate-2-sulfatase gene; IT, intrathecal; NA, notavailable; SD, standard deviation.

a For the safety population, baseline was the phase 2/3 study baseline for the early idursulfase-IT and sub-study groups, and the closest available value before the initial IDDD implantation surgery that took place in the extension for the delayed idursulfase-IT group. For the efficacy population, baseline was phase 2/3 study baseline.

b All patients in the extension study who underwent IDDD implantation surgery or received at least one dose of study drug (lull or partial).

c Separate sub-study for patients younger than 3 years at baseline.

d All patients in the extension study younger than 6 years at baseline who underwent IDDD implantation surgery or received at least one dose of study drug (full or partial).

**Table 2:** Summary of TEAEs^a^ by treatment group at month 36 (safety population^b^).

	Early idursulfase-IT (including sub-study^c^) (*n* = 41)		Delayed idursulfase-IT (*n* = 15)		Overall (*N* = 56)	
			
	Patients, *n* (%)	Events	Patients, *n* (%)	Events	Patients, *n* (%)	Events

At least one AE	41 (100.0)	2378	15 (100.0)	743	56 (100.0)	3121
At least one IV idursulfase infusion-related AE	12 (29.3)	24	2 (13.3)	7	14 (25.0)	31
At least one AE related to idursulfase-IT	33 (80.5)	308	11 (73.3)	87	44 (78.6)	395
At least one severe AE	14 (34.1)	26	7 (46.7)	14	21 (37.5)	40
At least one life-threatening AE	1 (2.4)	3	1 (6.7)	1	2 (3.6)	4
At least one IDDD-related AE	38 (92.7)	218	13 (86.7)	83	51 (91.1)	301
At least one SAE	33 (80.5)	108	10 (66.7)	29	43 (76.8)	137
Discontinued owing to an AE	0	0	0	0	0	0
Deaths	0	0	0	0	0	0

AE, adverse event; EOS, end of study; IDDD, intrathecal drug delivery device; IT, intrathecal; IV, intravenous; SAE, serious adverse event; TEAE, treatment-emergent adverse event.

a TEAEs are defined as all AEs occurring on or after the date of the first IDDD implantation surgery or first dose (whichever is earlier) and on or before the EOS visit (+ 30 days) or 2 weeks after the removal of the last IDDD (whichever is later).

b All patients in the extension study who underwent IDDD implantation surgery or received at least one dose of study drug (full or partial).

c Separate sub-study for patients younger than 3 years at baseline.

## Data Availability

The datasets, including the redacted study protocol, redacted statistical analysis plan and individual participants’ data supporting the results reported in this article, will be made available within 3 months from initial request to researchers who provide a methodologically sound proposal. The data will be provided after its de-identification in compliance with applicable privacy laws, data protection and requirements for consent and anonymization.

## Collaborators

The authors are grateful to the following investigators for their contributions to this study (listed alphabetically): Kyrieckos Aleck (Phoenix Children’s Hospital, Phoenix, AZ, USA), Hernán Amartino (Hospital Universitario Austral, Buenos Aires, Argentina), George Anadiotis (Randall Children’s Hospital at Legacy Emanuel, Portland, OR, USA), Paul Benke (Joe DiMaggio Children’s Hospital, Hollywood, FL, USA), Angelika Erwin (The Cleveland Clinic Foundation, Cleveland, OH, USA), Can Ficicioglu (Children’s Hospital of Philadelphia, Philadelphia, PA, USA), Dorothy Grange (Washington University School of Medicine, St Louis, MO, USA), Rabi Hanna (The Cleveland Clinic Foundation, Cleveland, OH, USA), Aneal Khan (Alberta Children’s Hospital, Calgary, AB, Canada), Martha Luz Solano Villarreal (Fundacion Cardioinfantil y LaCardio, Bogota, Colombia), Jim McGill (Queensland Children’s Hospital, South Brisbane, QLD, Australia), Damara Ortiz (Children’s Hospital of Pittsburgh, Pittsburgh, PA, USA), Helio Pedro (Hackensack University Medical Center, Hackensack, NJ, USA), William Rizzo (University of Nebraska Medical Center, Omaha, NE, USA), Devorah Segal (NYU Langone Medical Center, New York, NY, USA), Caroline Sevin (Hôpital Bicêtre, Le Kremlin Bicêtre, France), and Susan Spiller (East Tennessee Children’s Hospital, Knoxville, TN, USA), Angela Sun (Seattle Children’s Hospital, Seattle, WA, USA).

## Abbreviations:

ABC: Adaptive Behavior Composite
AE: adverse event
CI: confidence interval
CSF: cerebrospinal fluid
DAS-II: Differential Ability Scales-II
ECG: electrocardiogram
ERT: enzyme replacement therapy
GAG: glycosaminoglycan
GCA: General Conceptual Ability
HS: heparan sulfate
I2S: iduronate-2-sulfatase
IDDD: intrathecal drug delivery device
*IDS*: iduronate-2-sulfatase gene
IT: intrathecal
IV: intravenous
MMRM: mixed-effects model for repeated measures
MPS II: mucopolysaccharidosis II
NAb: neutralizing antibody
SAE: serious adverse event
SD: standard deviation
SE: standard error
TEAE: treatment-emergent adverse event
VABS-II: Vineland Adaptive Behavior Scales-II

## References

[1] Demydchuk M, Hill CH, Zhou A, Bunkoczi G, Stein PE, Marchesan D, Deane JE, Read RJ, Insights into Hunter syndrome from the structure of iduronate-2-sulfatase, Nat. Commun. 8 (2017) 15786. 28593992 10.1038/ncomms15786 PMC5472762

[2] Neufeld EF, Muenzer J, The mucopolysaccharidoses, in: Scriver CR, Beaudet AL, Sly WS, , (Eds.), The Metabolic and Molecular Bases of Inherited Disease, McGraw-Hill, New York 2001, pp. 3421–3452.

[3] Muenzer J, Giugliani R, Scarpa M, Tylki-Szymanska A, Jego V, Beck M, Clinical outcomes in idursulfase-treated patients with mucopolysaccharidosis type II: 3-year data from the hunter outcome survey (HOS), Orphanet J. Rare Dis. 12 (2017) 161. 28974237 10.1186/s13023-017-0712-3 PMC5627440

[4] Muenzer J, Beck M, Eng CM, Escolar ML, Giugliani R, Guffon NH, Harmatz P, Kamin W, Kampmann C, Koseoglu ST, Link B, Martin RA, Molter DW, Munoz Rojas MV, Ogilvie JW, Parini R, Ramaswami U, Scarpa M, Schwartz IV, Wood RE, Wraith E, Multidisciplinary management of Hunter syndrome, Pediatrics 124 (2009) e1228–e1239. 19901005 10.1542/peds.2008-0999

[5] Holt JB, Poe MD, Escolar ML, Natural progression of neurological disease in mucopolysaccharidosis type II, Pediatrics 127 (2011) e1258–e1265. 21518713 10.1542/peds.2010-1274

[6] Young ID, Harper PS, The natural history of the severe form of Hunter’s syndrome: a study based on 52 cases, Dev. Med. Child Neurol 25 (1983) 481–489. 6413286 10.1111/j.1469-8749.1983.tb13794.x

[7] Burton BK, Jego V, Mikl J, Jones SA, Survival in idursulfase-treated and untreated patients with mucopolysaccharidosis type II: data from the hunter outcome survey (HOS), J. Inherit. Metab. Dis. 40 (2017) 867–874. 28887757 10.1007/s10545-017-0075-x

[8] Seo JH, Okuyama T, Shapiro E, Fukuhara Y, Kosuga M, Natural history of cognitive development in neuronopathic mucopolysaccharidosis type II (Hunter syndrome): contribution of genotype to cognitive developmental course, Mol. Genet. Metab. Rep 24 (2020), 100630. 32775211 10.1016/j.ymgmr.2020.100630 PMC7394748

[9] Shapiro EG, Eisengart JB, The natural history of neurocognition in MPS disorders: a review, Mol. Genet. Metab. 133 (2021) 8–34. 33741271 10.1016/j.ymgme.2021.03.002

[10] Muenzer J, Wraith JE, Beck M, Giugliani R, Harmatz P, Eng CM, Vellodi A, Martin R, Ramaswami U, Gucsavas-Calikoglu M, Vijayaraghavan S, Wendt S, Puga AC, Ulbrich B, Shinawi M, Cleary M, Piper D, Conway AM, Kimura A, A phase II/III clinical study of enzyme replacement therapy with idursulfase in mucopolysaccharidosis II (Hunter syndrome), Genet. Med. 8 (2006) 465–473. 16912578 10.1097/01.gim.0000232477.37660.fb

[11] Muenzer J, Beck M, Eng CM, Giugliani R, Harmatz P, Martin R, Ramaswami U, Vellodi A, Wraith JE, Cleary M, Gucsavas-Calikoglu M, Puga AC, Shinawi M, Ulbrich B, Vijayaraghavan S, Wendt S, Conway AM, Rossi A, Whiteman DA, Kimura A, Long-term, open-labeled extension study of idursulfase in the treatment of Hunter syndrome, Genet. Med. 13 (2011) 95–101. 21150784 10.1097/GIM.0b013e3181fea459

[12] Muenzer J, Bodamer O, Burton B, Clarke L, Frenking GS, Giugliani R, Jones S, Rojas MV, Scarpa M, Beck M, Harmatz P, The role of enzyme replacement therapy in severe Hunter syndrome—an expert panel consensus, Eur. J. Pediatr. 171 (2012) 181–188. 22037758 10.1007/s00431-011-1606-3 PMC3249184

[13] Calias P, Papisov M, Pan J, Savioli N, Belov V, Huang Y, Lotterhand J, Alessandrini M, Liu N, Fischman AJ, Powell JL, Heartlein MW, CNS penetration of intrathecal-lumbar idursulfase in the monkey, dog and mouse: implications for neurological outcomes of lysosomal storage disorder, PLoS One 7 (2012), e30341. 22279584 10.1371/journal.pone.0030341 PMC3261205

[14] Muenzer J, Burton BK, Harmatz P, Gutiérrez-Solana LG, Ruiz-Garcia M, Jones SA, Guffon N, Inbar-Feigenberg M, Bratkovic D, Hale M, Wu Y, Yee KS, Whiteman DAH, Alexanderian D, Intrathecal idursulfase-IT in patients with neuronopathic mucopolysaccharidosis II: results from a phase 2/3 randomized study, Mol. Genet. Metab. 137 (2022) 127–139. 36027721 10.1016/j.ymgme.2022.07.017 PMC10826424

[15] Elliott C, Differential Ability Scales, Introductory and Technical Handbook, 2nd ed. Harcourt Assessment, San Antonio, TX, 2007.

[16] Muenzer J, Early initiation of enzyme replacement therapy for the mucopolysaccharidoses, Mol. Genet. Metab. 111 (2014) 63–72. 24388732 10.1016/j.ymgme.2013.11.015

[17] Muenzer J, Beck M, Giugliani R, Suzuki Y, Tylki-Szymanska A, Valayannopoulos V, Vellodi A, Wraith JE, Idursulfase treatment of Hunter syndrome in children younger than 6 years: results from the hunter outcome survey, Genet. Med. 13 (2011) 102–109. 21233716 10.1097/GIM.0b013e318206786f

[18] Broomfield A, Davison J, Roberts J, Stewart C, Hensman P, Beesley C, Tylee K, Rust S, Schwahn B, Jameson E, Vijay S, Santra S, Sreekantam S, Ramaswami U, Chakrapani A, Raiman J, Cleary MA, Jones SA, Ten years of enzyme replacement therapy in paediatric onset mucopolysaccharidosis II in England, Mol. Genet. Metab. 129 (2020) 98–105. 31383595 10.1016/j.ymgme.2019.07.016

[19] Zhang W, Xie T, Sheng H, Shao Y, Lin Y, Jiang M, Xu A, Su X, Liu Z, Zhao X, Liu L, Huang Y, Genetic analysis of 63 Chinese patients with mucopolysaccharidosis type II: functional characterization of seven novel IDS variants, Clin. Chim. Acta 491 (2019) 114–120. 30639582 10.1016/j.cca.2019.01.009

[20] Kosuga M, Mashima R, Hirakiyama A, Fuji N, Kumagai T, Seo JH, Nikaido M, Saito S, Ohno K, Sakuraba H, Okuyama T, Molecular diagnosis of 65 families with mucopolysaccharidosis type II (Hunter syndrome) characterized by 16 novel mutations in the IDS gene: genetic, pathological, and structural studies on iduronate-2-sulfatase, Mol. Genet. Metab. 118 (2016) 190–197. 27246110 10.1016/j.ymgme.2016.05.003

[21] Barbier AJ, Bielefeld B, Whiteman DA, Natarajan M, Pano A, Amato DA, The relationship between anti-idursulfase antibody status and safety and efficacy outcomes in attenuated mucopolysaccharidosis II patients aged 5 years and older treated with intravenous idursulfase, Mol. Genet. Metab. 110 (2013) 303–310. 23988379 10.1016/j.ymgme.2013.08.002

[22] Pano A, Barbier AJ, Bielefeld B, Whiteman DA, Amato DA, Immunogenicity of idursulfase and clinical outcomes in very young patients (16 months to 7.5 years) with mucopolysaccharidosis II (Hunter syndrome), Orphanet J. Rare Dis. 10 (2015) 50. 25902842 10.1186/s13023-015-0265-2 PMC4416269

