# Supplementary material for: Long-term open-label extension study of the safety and efficacy of intrathecal idursulfase-IT in patients with neuronopathic mucopolysaccharidosis II
Source: Mol Genet Metab. Author manuscript; Available in PMC 2024 Jan 30. (PMC10826456; doi:10.1016/j.ymgme.2022.07.016)
Supplement: Supplementary data [file NIHMS1948753-supplement-Supplementary_data.docx]

**Long-term open-label extension study of the safety and efficacy of intrathecal idursulfase-IT in patients with neuronopathic mucopolysaccharidosis II**

**Supplementary Materials**

Joseph Muenzer, Barbara K. Burton, Paul Harmatz, *et al.*, on behalf of the SHP609-302 study group

**Supplementary Table 1**. VABS-II ABC standard scores in patients younger than 6 years.

**A.** **Patients with missense *IDS* variants**

|  | **Month 13** | | **Month 24** | | **Month 36** | |
| --- | --- | --- | --- | --- | --- | --- |
|  | **Early idursulfase-IT (*n* = 13)** | **Delayed idursulfase-IT (*n* = 6)** | **Early idursulfase-IT (*n* = 13)** | **Delayed idursulfase-IT (*n* = 6)** | **Early idursulfase-IT (*n* = 13)** | **Delayed idursulfase-IT (*n* = 6)** |
| ***N*** | 12 | 6 | 11 | 5 | 11 | 3 |
| **VABS-II ABC score** |  |  |  |  |  |  |
| Least-squares mean (SE) | –4.0 (2.62) | –7.8 (3.72) | –7.5 (2.69) | –10.0 (3.93) | –10.0 (2.72) | –15.7 (4.47) |
| 90% CI | –8.5, 0.4 | –14.1, –1.4 | –12.1, –3.0 | –16.7, –3.4 | –14.6, –5.4 | –23.2, –8.1 |
| **Treatment difference** |  |  |  |  |  |  |
| Least-squares mean (SE) | 3.7 (4.56) | | 2.5 (4.77) | | 5.7 (5.25) | |
| 90% CI | –4.0, 11.5 | | –5.6, 10.6 | | –3.2, 14.5 | |
| *p* value | N/C | | N/C | | 0.2873 | |

**B. Patients with *IDS* variants other than missense**

|  | **Month 13** | | **Month 24** | | **Month 36** | |
| --- | --- | --- | --- | --- | --- | --- |
|  | **Early  idursulfase-IT (*n* = 13)** | **Delayed idursulfase-IT (*n* = 6)** | **Early idursulfase-IT (*n* = 13)** | **Delayed idursulfase-IT (*n* = 6)** | **Early  idursulfase-IT (*n* = 13)** | **Delayed idursulfase-IT (*n* = 6)** |
| ***N*** | 14 | 6 | 13 | 4 | 14 | 5 |
| **VABS-II ABC score** |  |  |  |  |  |  |
| Least-squares mean (SE) | –5.0 (2.94) | –0.3 (4.46) | –14.4 (2.96) | –4.1 (4.85) | –20.8 (2.94) | –24.1 (4.84) |
| 90% CI | –10.0, –0.1 | –7.8, 7.2 | –19.4, –9.4 | –12.2, 4.1 | –25.8, –15.9 | –32.2, –15.9 |
| **Treatment difference** |  |  |  |  |  |  |
| Least-squares mean (SE) | –4.7 (5.31) | | –10.3 (5.63) | | 3.3 (5.60) | |
| 90% CI | –13.7, 4.2 | | –19.8, –0.8 | | –6.2, 12.7 | |
| *p* value | N/C | | N/C | | 0.5634 | |

ABC, Adaptive Behavior Composite; CI, confidence interval; *IDS*, iduronate-2-sulfatase gene; N/C, not calculated; SE, standard error; VABS-II, Vineland Adaptive Behavior Scales-II.


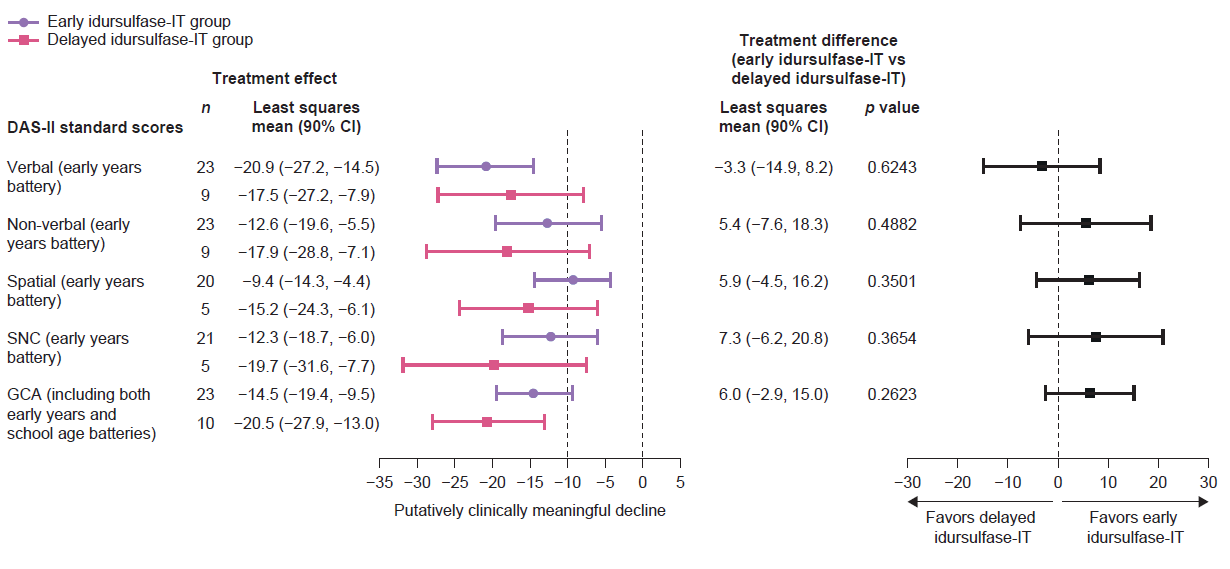


**Supplementary Fig. 1.** DAS-II cluster scores at month 36 in patients younger than 6 years at baseline.

CI, confidence interval; DAS-II, Differential Ability Scales-II; GCA, General Conceptual Ability; IT, intrathecal; SNC, special non-verbal composite.


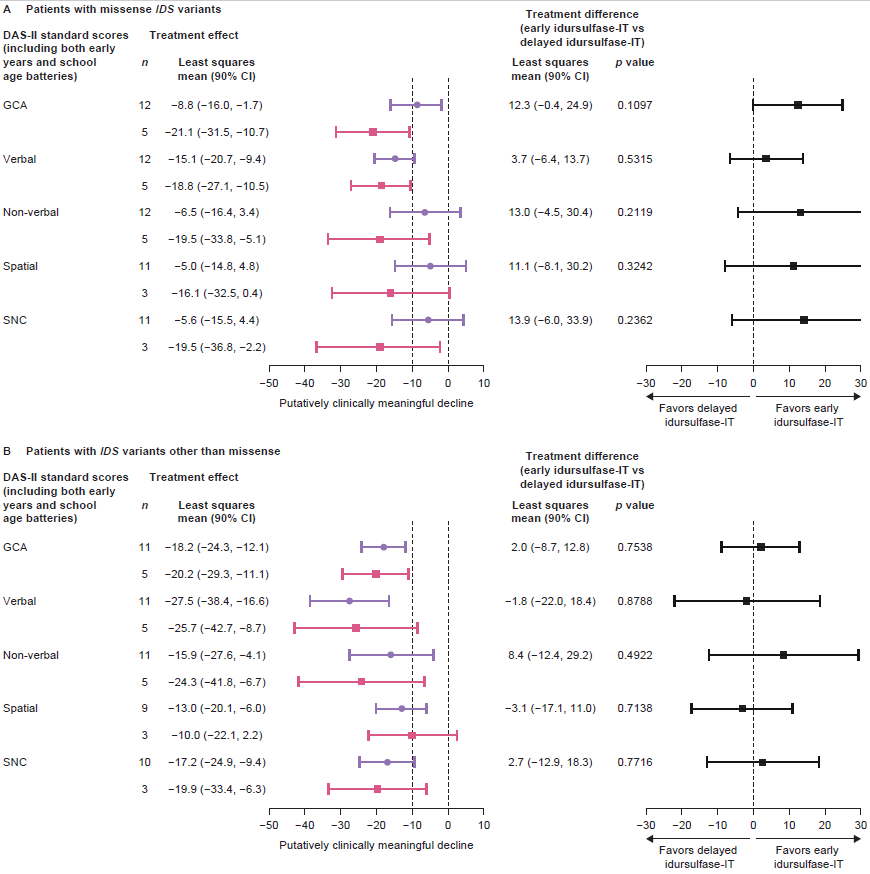


**Supplementary Fig. 2.** Treatment difference in DAS-II composite and cluster standard scores from MMRM models by *IDS* genotype at month 36 in patients younger than 6 years at baseline.

CI, confidence interval; DAS-II, Differential Ability Scales-II; GCA, General Conceptual Ability; *IDS*, iduronate-2-sulfatase gene; IT, intrathecal; MMRM, mixed-effects model repeated measures; SNC, special non-verbal composite.


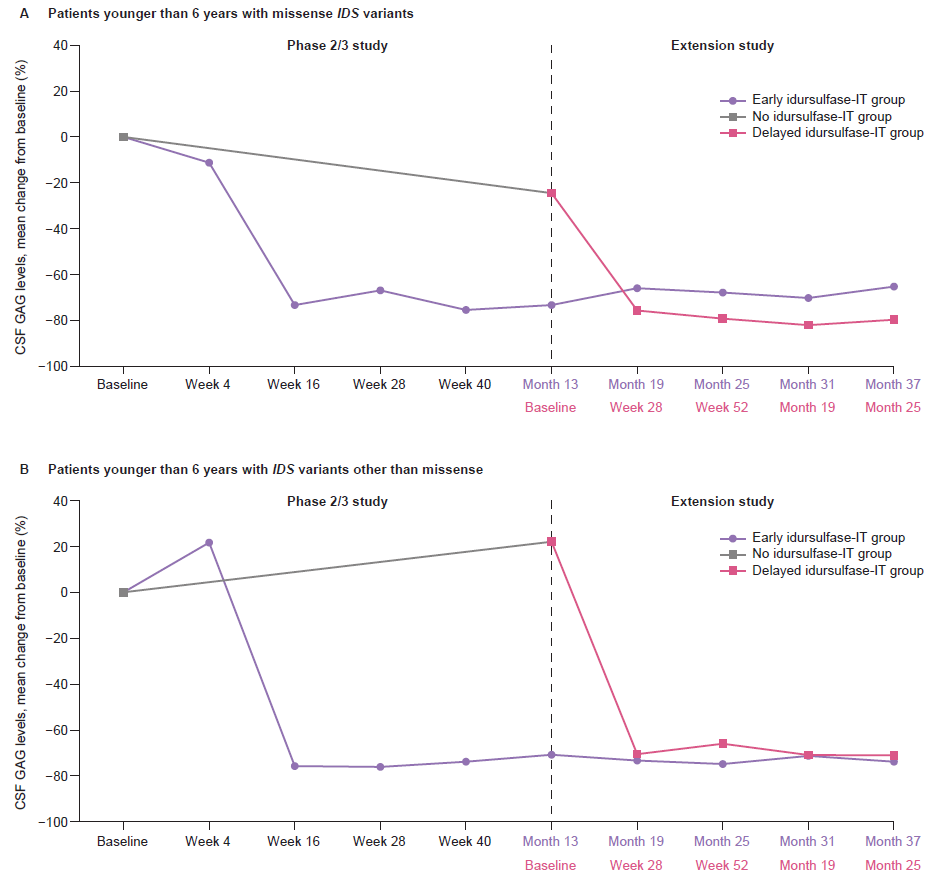


**Supplementary Fig. 3.** Mean percentage change from baseline in CSF total GAG levels in patients younger than 6 years at baseline with (A) missense *IDS* variants and (B) *IDS* variants other than missense.

CSF, cerebrospinal fluid; GAG, glycosaminoglycan; *IDS*, iduronate-2-sulfatase gene; IT, intrathecal.
